# Association Between Noninvasive Fibrosis Markers and Postoperative Mortality After Hepatectomy for Hepatocellular Carcinoma

**DOI:** 10.1001/jamanetworkopen.2018.7142

**Published:** 2019-01-18

**Authors:** Felipe B. Maegawa, Lauren Shehorn, Hassan Aziz, John Kettelle, Tun Jie, Taylor S. Riall

**Affiliations:** 1 Surgical Care Line, Southern Arizona Veterans Affairs Health Care System, Tucson; 2 Department of Surgery, University of Arizona, Tucson; 3 Quality Management, Robley Rex Veterans Affairs Medical Center, Louisville, Kentucky; 4 Department of Surgery, University of Southern California, Los Angeles

## Abstract

**Importance:**

The selection criteria for hepatectomy for hepatocellular carcinoma (HCC) is not well established. The role of noninvasive fibrosis markers in this setting is unknown in the US population.

**Objective:**

To evaluate whether aspartate aminotransferase–platelet ratio index (APRI) and fibrosis 4 (Fib4) values are associated with perioperative mortality and overall survival after hepatectomy for HCC.

**Design, Setting, and Participants:**

In a multicenter cohort study, Veterans Administration Corporate Data Warehouse was used to evaluate a retrospective cohort of 475 veterans who underwent hepatectomy for HCC between January 1, 2000, and December 31, 2012, in Veterans Administration hospitals. Data analysis occurred between September 30, 2016, and December 30, 2017. Logistic regression, survival analysis, and change in concordance index analysis were performed to evaluate the association between APRI and Fib4 values and mortality.

**Exposures:**

The cohort was stratified based on preoperative APRI and Fib4 values. Analysis was performed accounting for the validated and established predictors of outcome.

**Main Outcomes and Measures:**

Thirty-day mortality, 90-day mortality, and overall survival were the primary outcomes. An APRI value greater than 1.5 was considered high risk (cirrhosis), and an Fib4 value greater than 4.0 was considered high risk (advanced fibrosis). Portal hypertension (diagnosis of ascites or encephalopathy indicates presence of portal hypertension) and Child-Turcotte-Pugh (CTP) class (A indicates preserved liver function; B, mild to moderate liver dysfunction) served as 2 other measures of liver function.

**Results:**

A total of 475 patients with HCC underwent hepatectomy. The mean (SD) age was 65.6 (9.4) years; Model for End-Stage Liver Disease score, 8.9 (3.1); and body mass index, 28.1 (4.9) (calculated as weight in kilograms divided by height in meters squared). A total of 361 patients (76.0%) were men, 294 (61.9%) were white; 308 (64.8%) were hepatitis C positive, and 346 (72.8%) were categorized as CTP class A. The most common surgical procedure was partial lobectomy, with 321 (67.6%) procedures. The APRI value greater than 1.5 vs 1.5 or lower was associated with increased 30-day mortality (odds ratio [OR], 6.45; 95% CI, 2.80-14.80) and 90-day mortality (OR, 2.65; 95% CI, 1.35-5.22), as was Fib4 greater than 4.0 vs Fib4 4.0 or lower for 30-day mortality (OR, 5.41; 95% CI, 2.35-12.50) and 90-day mortality (OR, 2.74; 95% CI, 1.41-5.35). Survival analysis showed that overall survival was significantly different for APRI greater than 1.5 vs 1.5 or lower (mean survival time, 3.6 vs 5.4 years; log-rank *P* < .001) and Fib4 greater than 4.0 vs 4.0 or lower (mean survival time, 4.1 vs 5.3 years; log rank *P* = .01). Adjusted Cox proportional hazards regression analysis revealed that elevated APRI was significantly associated with worse survival (hazard ratio [HR], 1.13; 95% CI, 1.03-1.23) but Fib4 values were not (HR, 1.04; 95% CI, 0.99-1.09). Change in concordance index showed that APRI and Fib4 improved the ability of CTP class and portal hypertension to predict postoperative mortality.

**Conclusions and Relevance:**

Elevated APRI and Fib4 values, which are noninvasive markers of fibrosis, were associated with higher perioperative mortality. The APRI was also associated with worse overall survival. Use of APRI and Fib4 measures improved the ability of established markers to predict postoperative mortality. These findings suggest incorporating APRI and Fib4 to the selection process for hepatectomy for HCC as predictors associated with mortality may be warranted.

## Key Points

**Question:**

Are the noninvasive fibrosis markers aspartate aminotransferase–platelet ratio index and fibrosis 4 associated with perioperative mortality and overall survival after hepatectomy for hepatocellular carcinoma?

**Findings:**

In this cohort study of 475 US veterans, aspartate aminotransferase–platelet ratio index and fibrosis 4 were independently associated with increased 30- and 90-day mortality and worse overall survival. They were shown to improve the estimation of postoperative mortality.

**Meaning:**

This study suggests that the incorporation of aspartate aminotransferase–platelet ratio index and fibrosis 4 in the selection criteria of hepatectomy for hepatocellular carcinoma may be warranted.

## Teaser

This cohort study examines whether aspartate aminotransferase–platelet ratio index and fibrosis 4 values are asociated with perioperative mortality and overall survival among patients who undergo hepatectomy for hepatocellular carcinoma.

## Introduction

Hepatectomy and liver transplant are the main curative therapies for hepatocellular carcinoma (HCC).^1^ Intention-to-treat studies have demonstrated similar overall survival for these therapies.^2,3^ The selection criteria for HCC resection are not well established.^4^ The National Comprehensive Cancer Network guidelines for HCC state that patients with preserved liver function, resectable disease, who fit United Network for Organ Sharing criteria, could be considered for resection or transplant, and there remains controversy over which initial strategy is preferable.^1^ Hepatocellular carcinoma is currently the fastest rising cause of cancer-related deaths in the United States.^5^ Although the incidence of HCC tripled between 1975 and 2005, the 5-year survival rate remains approximately 25%.^6^ However, this survival rate could be greater than 70% when curative therapy is applied to early-stage HCC.^2,3,7,8^

Hepatic fibrosis is associated with an increased risk of postoperative liver failure and mortality after resection for HCC.^9^ The noninvasive fibrosis markers (NIFMs) aspartate aminotransferase–platelet ratio index (APRI) and fibrosis 4 (Fib4) have been shown to accurately predict the presence of cirrhosis and severe fibrosis when validated with liver biopsy findings.^10,11^ Studies conducted in Asian patients with hepatitis B and HCC demonstrated that abnormal levels of NIFMs were associated with worse postoperative outcomes after hepatectomy.^12,13,14^ However, to our knowledge, the role of these markers in the US population has not been studied. The aim of this study was to examine the association of APRI and Fib4 with perioperative mortality and overall survival after HCC resection among US veterans. We hypothesized these NIFMs independently associated with perioperative mortality and overall survival after hepatectomy for HCC.

## Methods

### Data Source and Study Cohort

The Veterans Administration (VA) Corporate Data Warehouse database was used. The Corporate Data Warehouse is a robust and comprehensive database that abstracts clinical information directly from the computerized patient record system from VA hospitals. Data were accessed, managed, and analyzed within the VA informatics and computing infrastructure. From January 1, 2000, to December 31, 2012, all patients with the diagnosis of HCC were identified using the *International Classification of Diseases, Ninth Revision, Clinical Modification* (*ICD-9-CM*) code 155.0 (liver cell carcinoma) and excluding patients with *ICD-9-CM* code 155.1 (intrahepatic bile duct carcinoma).^15^ We included all patients undergoing hepatectomy using the *Current Procedural Terminology* codes 47120 (partial lobectomy), 47125 (total left lobectomy), 47130 (total right lobectomy), and 47122 (trisegmentectomy). Data abstraction and management started September 30, 2016, and the analysis was completed on December 30, 2017.

The study protocol was reviewed and approved by the institutional review board of the Southern Arizona VA Health Care System, which also waived informed patient consent because this project was limited to retrospective deidentified data analysis. This study followed the Strengthening the Reporting of Observational Studies in Epidemiology (STROBE) reporting guideline.^16^

### Patient Demographics and Clinical Factors

Demographic, laboratory, and clinical data were ascertained at the time of resection. Race/ethnicity information was defined by the participants and was available on the CDW database. The preoperative laboratory data were obtained from the closest date to surgery. Liver function was determined using Model for End-Stage Liver Disease (MELD) score (score of 6-9 indicates normal liver function; ≥10 indicates liver dysfunction) and Child-Turcotte-Pugh (CTP) class (A indicates preserved liver function; B, mild to moderate liver dysfunction), as previously described.^17,18^ The presence of advanced fibrosis and cirrhosis was determined using the NIFMs Fib4 and APRI, respectively. Based on the studies that validated these NIFMs with liver biopsy and meta-analysis, the cutoff value for Fib4 was 4.0 (>4.0 indicates advanced fibrosis) and for APRI, 1.5 (>1.5 indicates cirrhosis).^10,11,19,20^ Standard equations were used:

Fib4 = [age (years) × AST level (U/L)] / [platelet count (10^9^) × √ALT level (U/L)], and

APRI = [AST level (U/L) / AST (upper limit of normal)] / platelet count (10^9^) × 100,

where ALT indicates alanine aminotransferase and AST indicates aspartate aminotransferase.

The main outcomes were defined as perioperative mortality and long-term survival; 30- and 90-day mortality were examined. Long-term survival was evaluated with overall survival comparison for patients with at least 1-month follow-up.

### Statistical Analysis

A 2-tailed, unpaired *t* test or Mann-Whitney test was used for univariate comparisons of continuous variables, and Pearson χ^2^ or Fisher exact test was used for comparison of categorical variables. A logistic regression model was used to evaluate the association between APRI and Fib4 and perioperative mortality. Overall survival was calculated from the date of surgery to date of death or last follow-up. The Kaplan-Meier method was used to plot the survival curves, and comparisons were performed with the log-rank test. Cox proportional hazards regression analysis was performed to evaluate the association between APRI and Fib4 levels with long-term survival. Previously established predictors of perioperative mortality were included in the multivariate models. To evaluate the predictive contribution of APRI and Fib4, their association with the concordance index of the predictive model composed of the established predictors of outcome (CTP and portal hypertension) were examined as previously described.^21^ Complete case analysis was used for missing data.

Statistical comparisons were 2-sided, and *P* values <.05 were considered significant. The statistical analysis was conducted using the SAS Enterprise Guide, version 7.1 (SAS Institute Inc).

## Results

### Patient and Operative Characteristics of the Whole Cohort

A total of 11 497 veterans were diagnosed with HCC between January 1, 2000, and December 31, 2012. Of those, 475 patients (4.1%) underwent liver resection. The clinical characteristics of the whole cohort are outlined in Table 1. The mean (SD) age was 65.6 (9.4) years, 361 (76.0%) were men, and 294 (61.9%) were white. Hepatitis C, present in 308 patients (64.8%), was the most common cause of liver disease. Partial lobectomy was performed in 321 patients (67.6%), whereas major hepatectomy was performed in 154 patients (32.4%). As expected for surgical patients, most had neither ascites (349 [73.5%]) nor encephalopathy (420 [88.4%]), and CTP class A was the most common level (346 [72.8%]). The mean (SD) values were 8.9 (3.1) for MELD, 1.1 (1.3) for APRI, and 3.4 (2.7) for Fib4. The mean follow-up period was 4.7 (3.8) years. The 30- and 90-day mortality rates were 5.9% and 10.1%, respectively. The median overall survival was 3.9 (95% CI, 3.3-4.5) years.

**Table 1.** Baseline Clinical Characteristics of 475 Patients Who Underwent Liver Resection for Hepatocellular Carcinoma

Variable	Patients (N = 475)
Age, mean (SD), y	65.6 (9.4)
Sex, No. (%)	
Women	8 (1.7)
Men	361 (76.0)
Missing	106 (22.3)
Race, No. (%)	
White	294 (61.9)
Other	143 (30.1)
Missing	38 (8.0)
BMI, mean (SD)	28.1 (4.9)
Cirrhosis cause, No. (%)	
Non-HCV	167 (35.2)
HCV	308 (64.8)
Hepatectomy type, No. (%)	
Major lobectomy (right, left, trisegmentectomy)	154 (32.4)
Partial lobectomy	321 (67.6)
Bilirubin, mean (SD), mg/dL	1.13 (0.4)
Albumin, mean (SD), g/dL	3.97 (3.2)
Missing, No. (%)	22 (4.6)
Creatinine, mean (SD), mg/dL	1.17 (0.48)
INR, mean (SD)	1.11 (0.1)
Ascites, No. (%)	
No	349 (73.5)
Yes	126 (26.5)
Encephalopathy, No. (%)	
No	420 (88.4)
Yes	55 (11.6)
CTP class, mean (SD), points^a^	5.9 (1.1)
A	346 (72.8)
B	129 (27.2)
MELD score, mean (SD)^b^	8.9 (3.1)
APRI, mean (SD)^c^	1.1 (1.3)
Fib4, mean (SD)^d^	3.4 (2.7)
Follow-up, mean (SD), y	4.7 (3.8)
Mortality, No. (%)	
30-Day	28 (5.9)
90-Day	48 (10.1)
Overall survival, y	
Mean (SE)	5.1 (0.2)
Median (95% CI)	3.9 (3.3-4.5)

Abbreviations: APRI, aspartate aminotransferase–platelet ratio index; BMI, body mass index (calculated as weight in kilograms divided by height in meters squared); CTP, Child-Turcotte-Pugh; Fib4, fibrosis 4; HCV, hepatitis C virus; INR, international normalized ratio; MELD, Model for End-Stage Liver Disease.

SI conversion units: To convert albumin to grams per liter, multiply by 10; bilirubin to micromoles per liter, multiply by 17.104; creatinine to micromoles per liter, multiply by 88.4.

^a^ CTP A indicates preserved liver function; B, mild to moderate liver dysfunction.

^b^ MELD score of 6 to 9 indicates normal liver function; 10 or higher indicates liver dysfunction.

^c^ An APRI value greater than 1.5 was considered high risk (cirrhosis).

^d^ An Fib4 value greater than 4.0 was considered high risk (advanced fibrosis).

### APRI and Fib4 Association With Perioperative Mortality and Survival 

Logistic regression was performed to examine the association between APRI and Fib4 and the 30- and 90-day mortality. Compared with patients with APRI values 1.5 or lower, multivariable analysis revealed that APRI greater than 1.5 was associated with worse 30-day (OR, 6.45; 95% CI, 2.80-14.80; *P* < .001) and 90-day (OR, 2.65; 95% CI, 1.35-5.22; *P* = .005) mortality. Likewise, multivariable analysis showed that, compared with Fib4 levels of 4.0 or lower, Fib4 levels greater than 4.0 were associated with worse 30-day (OR, 5.41; 95% CI, 2.35-12.50; *P* < .001) and 90-day (OR, 2.74; 95% CI, 1.41-5.35; *P* = .003) mortality (Table 2).

**Table 2.** Multivariable Logistic Regression Analysis of APRI and Fib4 for 30- and 90-Day Mortality After Hepatectomy for Hepatocellular Carcinoma

Variable	Category	30-d Mortality		90-d Mortality	
		OR (95% CI)	*P* Value	OR (95% CI)	*P* Value
APRI^a^	>1.5	6.45 (2.80-14.8)	<.001	2.65 (1.35– 5.22)	.005
CTP class^b^	B	3.56 (1.37-9.30)	.009	3.51 (1.64-7.51)	<.001
Portal hypertension	Present	7.27 (2.20-24.1)	.001	2.74 (1.20-6.28)	.01
Fib4^c^	>4.0	5.41 (2.35-12.5)	<.001	2.74 (1.41-5.35)	.003
CTP class^b^	B	3.53 (1.35-9.17)	.009	3.41 (1.58-7.34)	.002
Portal hypertension	Present	7.18 (2.17-23.7)	.001	2.82 (1.22-6.51)	.01

Abbreviations: APRI, aspartate aminotransferase–platelet ratio index; CTP, Child-Turcotte-Pugh; Fib4, fibrosis 4; OR, odds ratio.

^a^ An APRI value greater than 1.5 was considered high risk (cirrhosis).

^b^ CTP class B was considered high risk (liver dysfunction).

^c^ An Fib4 value greater than 4.0 was considered high risk (advanced fibrosis).

**Figure 1.**
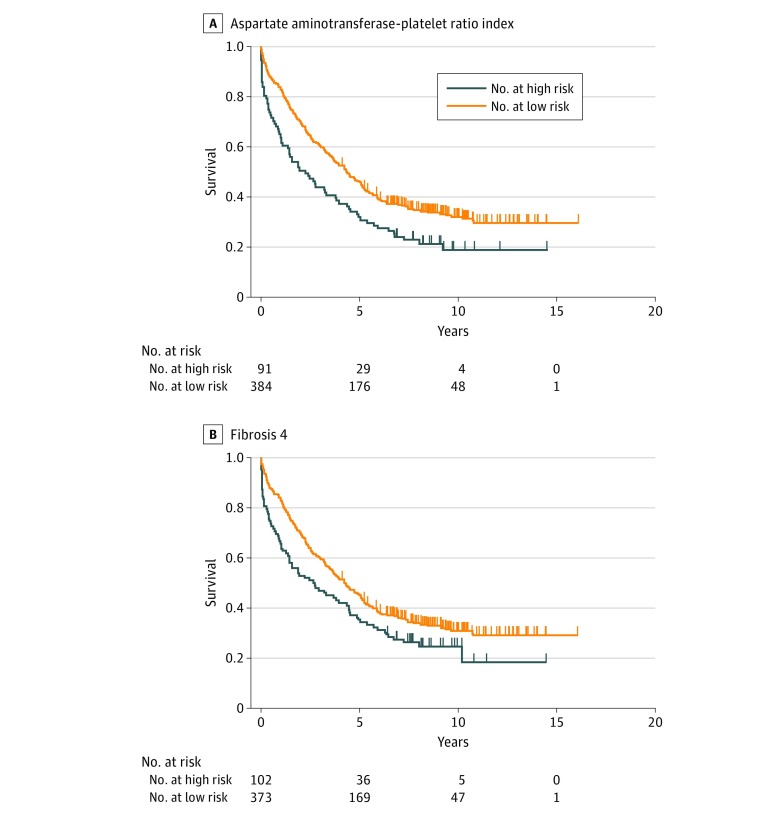
Survival of Patients Undergoing Hepatectomy for Hepatocellular Carcinoma A, Patients stratified by aspartate aminotransferase–platelet ratio index. High risk was considered an aspartate aminotransferase–platelet ratio index value greater than 1.5 (cirrhosis); low risk, aspartate aminotransferase–platelet ratio index 1.5 or lower (log-rank test *P* < .001). B, Patients stratified by fibrosis 4. High risk was considered a fibrosis 4 value greater than 4.0 (advanced fibrosis); low risk, fibrosis 4 value of 4.0 or lower (log-rank test *P* = .01).

Kaplan-Meier survival analysis showed that patients with APRI levels greater than 1.5 had worse overall survival than patients with APRI levels of 1.5 or lower (mean survival time, 3.6 vs 5.4 years; log-rank test *P* < .001) (Figure 1A). The median survival for patients with APRI greater than 1.5 and APRI 1.5 or less were 2.2 and 4.3 years, respectively. When patients were stratified by Fib4 index, the overall survival between patients with Fib4 greater than 4.0 was significantly worse than patients with Fib4 of 4.0 or lower (mean survival, 4.1 vs 5.3 years; log-rank test *P* = .01) (Figure 1B). The median survival for patients with Fib4 greater than 4.0 and 4.0 or lower were 2.7 and 4.3 years, respectively. Multivariate Cox proportional hazards regression model analysis was conducted. After adjusting for CTP and the presence of portal hypertension, APRI was associated with worse overall survival after hepatectomy for HCC (hazard ratio [HR], 1.13; 95% CI, 1.03-1.23; *P* = .007). However, Fib4 was not significantly associated with worse overall survival (HR, 1.04; 95% CI, 0.99-1.09; *P* = .06).

### Change in the Concordance Index and APRI and Fib4

Concordance index is similar to the area under the receiver operating characteristic (ROC) curve and measures the probability that, given 2 random patients, the one with the worse outcome is predicted to have a worse outcome.^22^ To evaluate whether APRI and Fib4 could more accurately predict the postoperative mortality and overall survival after hepatectomy for HCC, these NIFMS were added to the model containing the established predictors: CTP class and portal hypertension. Table 3 reports 3 regression models for the prediction of 30- and 90-day mortality and overall survival. Each of the 3 models lacks 1 variable, and they are compared with the full model containing all variables (reference model). The predictive accuracy of 30-day mortality was improved by 0.12 (*P* = .009) when APRI was incorporated into the established model (CTP and portal hypertension). Likewise, the ability to predict 90-day mortality was improved by adding APRI to the established model by 0.05 (*P* = .01).

**Table 3.** Concordance Index Quantifying the Reduction in the Predictive Ability of the Model

Concordance Index Model	30-d Mortality Concordance Index		90-d Mortality Concordance Index		Long-term Survival Concordance Index	
	Decrease (95% CI)	*P* Value	Decrease (95% CI)	*P* Value	Decrease (95% CI)	*P* Value
APRI^a^						
CTP,^b^ portal hypertension, APRI	Full model [reference]	NA	Full model [reference]	NA	Full model [reference]	NA
CTP, APRI	0.06 (−0.01 to 0.15)	.13	0.008 (−0.02 to 0.04)	.67	0.11 (0.08 to 0.15)	<.001
Portal hypertension, APRI	0.01 (−0.03 to 0.06)	.59	0.04 (−0.03 to 0.12)	.26	0.08 (0.06 to 0.12)	<.001
CTP, portal hypertension	0.12 (0.03 to 0.22)	.009	0.05 (0.01 to 0.10)	.01	0.18 (0.08 to 0.29)	<.001
Fib4^c^						
CTP, portal hypertension, Fib4	Full model [reference]	NA	Full model [reference]	NA	Full model [reference]	NA
CTP, Fib4	0.03 (−0.03 to 0.10)	.35	0.006 (−0.03 to 0.04)	.74	0.12 (0.10 to 0.15)	<.001
Portal hypertension, Fib4	0.001 (−0.01 to 0.01)	.89	0.03 (−0.02 to 0.09)	.20	0.10 (0.07 to 0.12)	<.001
CTP, portal hypertension	0.13 (0.04 to 0.22)	.004	0.07 (0.01 to 0.14)	.01	0.17 (0.06 to 0.29)	.003

Abbreviations: APRI, aspartate aminotransferase–platelet ratio index; CTP, Child-Turcotte-Pugh; Fib4, fibrosis 4; NA, not applicable.

^a^ An APRI value greater than 1.5 was considered high risk (cirrhosis).

^b^ CTP class B was considered high risk (liver dysfunction).

^c^ An Fib4 value greater than 4.0 was considered high risk (advanced fibrosis).

**Figure 2.**
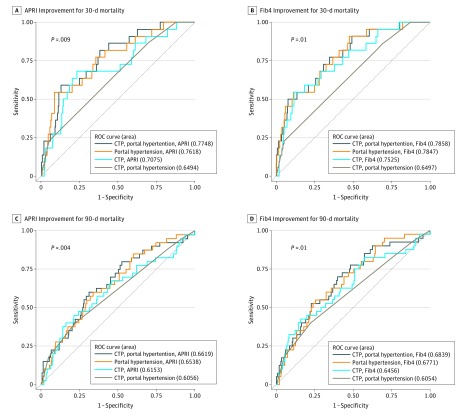
Sensitivity and Specificity of Aspartate Aminotransferase–Platelet Ratio Index (APRI) and Fibrosis 4 (Fib4) as Indicators of Mortality Receiver operating characteristic (ROC) curves with associated areas under the curve. A and B, APRI and Fib4 improvement of the predictive ability of the models for 30-day mortality. C and D, APRI and Fib4 improvement of the predictive ability of the models for 90-day mortality. *P* values represent the differences in areas under of the curve between the full model vs the model without APRI or Fib4 (Child-Turcotte-Pugh [CTP], portal hypertension). An APRI value greater than 1.5 was considered high risk (cirrhosis), and an Fib4 value greater than 4.0 was considered high risk (advanced fibrosis).

In addition, APRI improved the overall survival prediction by 0.18 (*P* < .001). Table 3 also demonstrates the association between Fib4 and the prediction accuracy of perioperative mortality and overall survival. The improvement of the predictive accuracy of 30- and 90-day mortality by adding Fib4 to the model was 0.13 (*P* = .004) and 0.07 (*P* = .01), respectively. The long-term survival prediction was improved by 0.17 (*P* = .003). Figure 2 illustrates the ROC curves and their respective areas under the curve, demonstrating the association between APRI and Fib4 with the predictive accuracy of 30- and 90-day mortality in patients after hepatectomy for HCC.

## Discussion

To our knowledge, this is the largest Western series to examine the role of NIFMs in patients undergoing hepatectomy for HCC. This study demonstrates the association of APRI and Fib4 with the perioperative mortality and overall survival after hepatectomy for HCC. Using cutoff values described and validated by previous studies, both APRI and Fib4 were independently associated with increased 30- and 90-day mortality.^10,11,20,23^ In addition, APRI was associated with worse overall survival, after adjusting for CTP class and portal hypertension. Child-Turcotte-Pugh classification and portal hypertension are the most validated and established predictors of outcome after liver resection, and are broadly used for selection of patients for liver resection for HCC.^24^ The perioperative mortality rate within 90 days from surgery for the 475 patients was found to be 10.1%, highlighting the need for better predictors of postoperative outcomes.

The present study is unique for being what we believe to be the first to demonstrate how APRI and Fib4 contribute to the ability to predict the perioperative mortality and overall survival beyond what is achieved by the established predictors. This improvement indicated by APRI and Fib4 was demonstrated using the concordance index change method.^21^ The decrease in the concordance index associated with the addition of APRI or Fib4 to the regression models was statistically significant for the prediction of 30- and 90-day mortality and overall survival. These findings suggest that both APRI and Fib4 contribute to the prediction of short- and long-term survival outcomes after resection of HCC, beyond what is achieved by CTP and portal hypertension.

This study supports the findings of other authors that APRI is independently associated with poor overall survival after liver resection for HCC.^12,13,14^ Hung et al^12^ and Shen et al^13^showed that APRI was associated with worse disease-free and overall survival. Hung et al^12^ evaluated APRI as a surrogate marker of hepatic fibrosis in 76 patients with hepatitis B who underwent hepatectomy for solitary and small HCC and reported that APRI was a reliable marker for assessing fibrosis and predicting survival. More recently, Shen et al, examining a cohort of 332 patients, reported that APRI was associated with worse disease-free and overall survival. The populations of these studies were predominantly patients with hepatitis B.^12,13^ A study from Japan, examining a large cohort of patients with hepatitis C, evaluated the association between APRI and postoperative liver failure. The investigators demonstrated that APRI was independently associated with an increased risk of postoperative liver failure.^14^

To our knowledge, the present study is the first to report the association between APRI and worse 30- and 90-day postoperative mortality. Furthermore, this appears to be the first report of the association of Fib4 with 30- and 90-day mortality after hepatectomy for HCC. Dong et al^23^ examined the association of Fib4 with postoperative complications and intraoperative blood loss in a large cohort of patients with hepatitis B (n = 350). They demonstrated that Fib4 was independently associated with adverse postoperative outcomes. Toyoda et al^25^ evaluated the association of Fib4 and long-term outcome after resection for HCC in a large series of 431 patients. They found that Fib4 was associated with worse disease-free and overall survival. These findings corroborate the results of our analysis. However, the present analysis differs from these previous studies because it was based on a large cohort of US veterans. The main cause of liver disease in these patients was hepatitis C. Moreover, this study appears to be the first to evaluate the predictive ability of APRI and Fib4 and their contribution to the established predictors of liver resection outcomes in an organized and methodologic manner.

For patients with CTP A without portal hypertension with resectable HCC, there is still controversy on which is the preferable initial strategy: liver resection or transplantation. The optimal selection criteria for these therapies have yet to be established.^1^ In this analysis, 4.1% of the patients with HCC underwent liver resection, which is consistent with previous studies on HCC treatment. Davila et al,^26^ also using the VA Hepatitis C Clinical Case Registry, reported that 4.4% of veterans with HCC underwent liver resection. Forner et al^24^ reported that only of 5% to 10% of patients are candidates for hepatectomy, because most centers restrict surgery to patients with good performance status, a single tumor, preserved liver function, and the absence of portal hypertension and vascular invasion. Child-Turcotte-Pugh class and portal hypertension are integral parts of the Barcelona Clinic Liver Cancer Staging System, which is the standard of care for HCC management in Western countries.^24^ Therefore, CTP class and portal hypertension were used in our analysis. The outcomes after hepatectomy for HCC have improved over the past decades, given better surgical techniques and perioperative care.^27,28^ Patient selection has played a significant role in reducing perioperative morbidity and mortality. Therefore, the Barcelona Clinic Liver Cancer Staging System could be further refined.^24^

Noninvasive fibrosis markers, such as APRI and Fib4, are attractive markers because they are easily accessible and determined with routine preoperative laboratory tests. Another unique characteristic of APRI and Fib4 is that they include the platelet count, which is useful because a low platelet count has been shown to be associated with increased perioperative morbidity and mortality.^29,30^ In addition, APRI and Fib4 are NIFMs that were validated prospectively with liver biopsy.^10,11^ Wai et al^11^ demonstrated that APRI can predict cirrhosis with an area under the ROC curve of 0.94. Sterling et al^10^ evaluated the discriminative ability of Fib4 in predicting liver fibrosis, reporting an area under the ROC curve of 0.756 for severe fibrosis. Therefore, APRI and Fib4 are good predictors of cirrhosis and fibrosis, respectively. Liver fibrosis and cirrhosis have been described as markers of impaired hepatic regeneration and increased risk of postoperative liver failure after hepatectomy.^9,31^ These findings, associated with the results of the present study, indicate that APRI and Fib4 are potential predictors of postoperative mortality after hepatectomy for HCC.

### Limitations

This study has several limitations, which are inherent to its observational retrospective design. Some unmeasured patient characteristics could have changed the results, in particular, the lack of information on preoperative comorbidities. However, the large number of patients of this cohort and the analysis accounting for the validated predictors of surgical outcomes helped to minimize possible selection bias. The VA CDW database constitutes predominately white, male patients. Therefore, the results of this study may not be generalizable to the overall population. Information on HCC recurrence and specific cause of death were not available. In addition, information on postoperative complications was not ascertained. Therefore, it is unknown whether perioperative mortality was related to liver failure or other causes.

## Conclusions

This study suggests that APRI greater than 1.5 and Fib4 greater than 4.0 were independently associated with increased 30- and 90-day mortality after hepatectomy for HCC. In addition, APRI was independently associated with worse overall survival. The contribution of APRI and Fib4 to improve the ability of established markers in predicting perioperative mortality and overall survival was supported by valid methods. These findings suggest that incorporating APRI and Fib4 in the selection process for hepatectomy for HCC as new predictors of mortality may be warranted.
